# The Structure and Function of Ionotropic Receptors in *Drosophila*

**DOI:** 10.3389/fnmol.2020.638839

**Published:** 2021-02-01

**Authors:** Lina Ni

**Affiliations:** School of Neuroscience, Virginia Tech, Blacksburg, VA, United States

**Keywords:** *Drosophila*, ionotropic receptors, chemosensation, gustation, olfaction, thermosensation, hygrosensation

## Abstract

Ionotropic receptors (IRs) are a highly divergent subfamily of ionotropic glutamate receptors (iGluR) and are conserved across Protostomia, a major branch of the animal kingdom that encompasses both Ecdysozoa and Lophothrochozoa. They are broadly expressed in peripheral sensory systems, concentrated in sensory dendrites, and function in chemosensation, thermosensation, and hygrosensation. As iGluRs, four IR subunits form a functional ion channel to detect environmental stimuli. Most IR receptors comprise individual stimulus-specific tuning receptors and one or two broadly expressed coreceptors. This review summarizes the discoveries of the structure of IR complexes and the expression and function of each IR, as well as discusses the future direction for IR studies.

## Introduction

Ionotropic receptors (IRs) are a highly divergent subfamily of ionotropic glutamate receptors (iGluR) (Benton et al., 2009). Most iGluRs bind the excitatory neurotransmitter glutamate and function in synaptic communication in the brain (Mayer and Armstrong, 2004). In contrast, IRs are primarily and broadly expressed in peripheral sensory systems and have diverse functions, including chemosensation, thermosensation, and hygrosensation (van Giesen and Garrity, 2017). They might also be involved in hearing and social cluster formation (Senthilan et al., 2012; Jiang et al., 2020). IRs are conserved across Protostomia, a major branch of the animal kingdom that encompasses both Ecdysozoa and Lophothrochozoa, and play a key role in host-seeking behavior in disease vectors, such as mosquitoes (Croset et al., 2010; Raji et al., 2019; Greppi et al., 2020; Jové et al., 2020). While “antennal” IRs are conserved across insects, often contain many introns, and function in olfaction, thermosensation, and hygrosensation; species-specific “divergent IRs” are often single intron genes, are expressed in peripheral and internal gustatory neurons, and are required for taste and food assessment (Benton et al., 2009; Croset et al., 2010).

As iGluRs, four IR subunits form a functional ion channel to allow cations–mainly monovalent cation, but also calcium–to flux into and activate sensory neurons (Abuin et al., 2011, 2019). Most IR receptors comprise individual stimulus-specific tuning receptors and one or two broadly expressed coreceptors (Abuin et al., 2011). IR proteins are detected in the cell body and sensory dendrites but not at synapses (Benton et al., 2009; Ai et al., 2010, 2013; Abuin et al., 2011; Grosjean et al., 2011). Expression of IRs in sensory dendrites is critical for sensory responses and requires the heteromeric IR complex formation (Abuin et al., 2011; Ai et al., 2013).

There are 63 IR proteins in *Drosophila melanogaster*, including four coreceptors (IR8a, IR25a, IR76b, and IR93a) and 59 tuning receptors (Benton et al., 2009; Koh et al., 2014; van Giesen and Garrity, 2017). The expression of most IRs–except for five tuning receptors (IR48a, IR51a, IR54a, IR60a, and IR87a)–has been described (Supplementary Tables 1, 2). However, the function of only 18 tuning receptors has been found (Table 1). In this review, I will first summarize the discoveries of the structure of IR complexes. Next, I will discuss the known function of IR proteins, including the discoveries from most recent studies as well as the information described in previous review articles (Rytz et al., 2013; van Giesen and Garrity, 2017; Rimal and Lee, 2018). Importantly, the information about IR expression and function, with references, will be listed in Table 1 and Supplementary Tables 1, 2. Finally, I will discuss the future direction for IR studies in *Drosophila melanogaster* and other animals.

**Table 1:** The known function of each IR.

**Tuning IR**	**Coreceptor**	**Function**	**Stimulus**	**Expression**	**References**
7a		Gustation	acetic acid	Labellum: taste bristles	Croset et al., 2010; Rimal et al., 2019
20a	76b	Gustation	amino acids	Tarsus: taste bristles	Ganguly et al., 2017
21a	25a/93a	Thermosensation	cool	Arista/DOG	Benton et al., 2009; Ni et al., 2016; Budelli et al., 2019
31a	8a	Olfaction	2-oxopentanoic acid	Antenna: sensilla	Benton et al., 2009; Silbering et al., 2011
40a	25a/93a	Hygrosensation	dry	Sacculus (I, II)	Benton et al., 2009; Enjin et al., 2016; Knecht et al., 2016
41a	25a/76b	Olfaction	pyridine, pyrrolidine, 1,4-diaminobutane (putrescine), cadaverine, and spermidine	Antenna: sensilla	Abuin et al., 2011; Silbering et al., 2011; Hussain et al., 2016
56d	25a/76b	Gustation	hexanoic acid and octanoic acid	Labellum: taste bristles and pegs; tarsus: taste bristles	Ahn et al., 2017; Tauber et al., 2017; Sanchez-Alcaniz et al., 2018
56d	25a/76b	Gustation	carbonation	Labellum: taste peg	Sanchez-Alcaniz et al., 2018
60b		Gustation	sucrose	Pharynx: LSO	Joseph et al., 2017
62a	25a/76b	Gustation	calcium	labellum	Lee et al., 2018
64a	8a	Olfaction	hydrochloride acid and acetic acid	Sacculus (III)	Ai et al., 2010, 2013; Silbering et al., 2011; Gao et al., 2015
68a	25a/93a	Hygrosensation	moist	Sacculus (II)	Frank et al., 2017; Knecht et al., 2017
75a	8a	Olfaction	acetic acid and propionic acid	Antenna: sensilla	Benton et al., 2009; Abuin et al., 2011; Silbering et al., 2011; Gorter et al., 2016; Prieto-Godino et al., 2016, 2017
75b/c	8a	Olfaction	propionic acid, butyric acid and 2-oxyopentanoic acid	Antenna: sensilla	Benton et al., 2009; Abuin et al., 2011; Silbering et al., 2011; Prieto-Godino et al., 2017
75d	25a	Olfaction	pyrrolidine	Antenna: sensilla	Benton et al., 2009; Silbering et al., 2011
76a	25a/76b	Olfaction	pyrrolidine and phenylethylamine	Antenna: sensilla	Benton et al., 2009; Abuin et al., 2011; Silbering et al., 2011
84a	8a	Olfaction	phenylacetic acid and phenylacetaldehyde	Antenna: sensilla	Benton et al., 2009; Grosjean et al., 2011; Silbering et al., 2011; Hueston et al., 2016
92a		Olfaction	ammonia, methylamine, dimethylamine, trimethylamine, ethylamine, triethylamine, butylamine and pyrrolidine	Antenna: sensilla	Benton et al., 2009; Abuin et al., 2011; Silbering et al., 2011; Min et al., 2013

## Structure

As iGluRs, four IR subunits are predicted to form a functional ion channel. This possibility has been confirmed by photobleaching and counting GFP-tagged subunits *in vitro* and analogy with the heterotetrameric stoichiometry of iGluRs (Abuin et al., 2011, 2019). For IR complexes that use IR8a as coreceptors, there are two IR8a subunits and two tuning IRs in each heterotetrameric complex (Abuin et al., 2011, 2019). Although IRs are iGluR-related proteins, they are not closely related to the well-described AMPA, NMDA, or kainate classes of iGluRs (Benton et al., 2009). Instead, the IR family is extremely divergent and the overall amino acid sequence identity is 10–70% (Benton et al., 2009).

**Figure 1:**
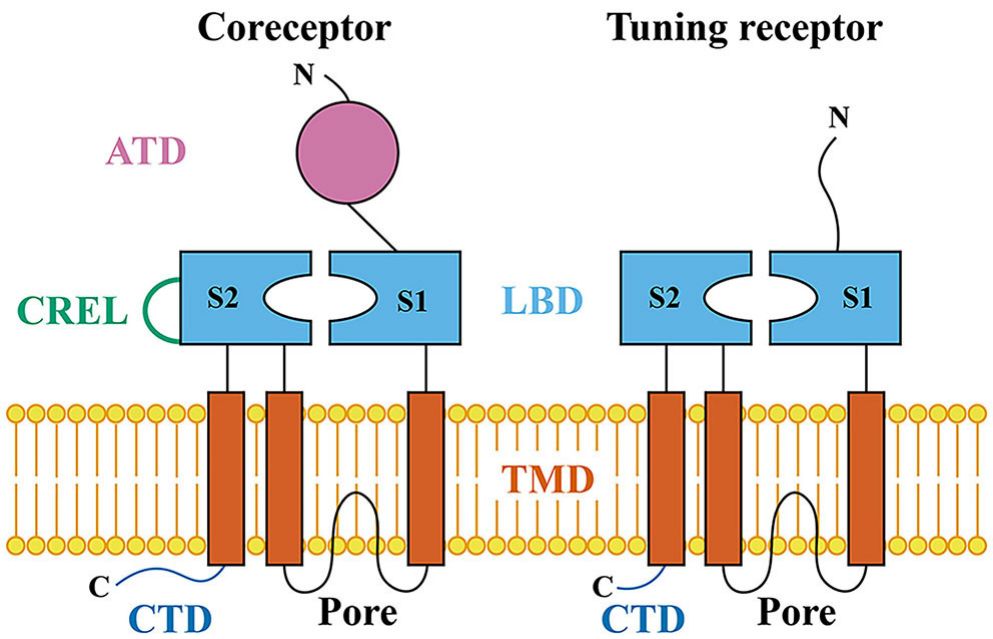
Schematic of the IR coreceptor and tuning receptor. ATD, the amino-terminal domain; LBD, the ligand-binding domain; S1 and S2, two half-domains of LBD that form a “Venus flytrap” structure; CREL, the coreceptor extra loop; TMD, the transmembrane domain; Pore, the ion channel pore region; CTD, the carboxy-terminal domain.

iGluRs contain four protein domains: the amino-terminal domain (ATD), ligand-binding domain (LBD), transmembrane domain (TMD), and carboxy-terminal domain (CTD) (Mayer and Armstrong, 2004). The extracellular ATD domain is followed by the LBD domain that contains two half-domains, S1 and S2. S1 and S2 form a “Venus flytrap” structure that closes upon binding of glutamate (Armstrong et al., 1998). In the primary structure, S1 and S2 are separated by the ion channel pore. The ion channel pore is formed by two transmembrane segments (TM1 and TM2) and a re-entrant pore loop (Kuner et al., 2003). S2 is followed by a third transmembrane segment (TM3) and a cytosolic carboxy-terminal domain (CTD). The IR coreceptors, IR8a and IR25a, contain the ATD, LBD, TMD, CTD, and coreceptor extra loop (CREL). Tuning IRs and less broadly expressed coreceptors, IR76b and IR93a, do not contain typical ATD domains and CRELs (Figure 1) (Benton et al., 2009; Croset et al., 2010).

The ATD domain is involved in the assembly of iGluR subunits into heteromeric complexes (Jin et al., 2009). The coreceptors, IR8a and IR25a, contain ATD domains (Figure 1) (Croset et al., 2010). Deletion of the ATD in IR8a abolishes the cilia localization, suggesting a role for this domain in protein folding, IR complex assembly, or cilia targeting (Abuin et al., 2011). Moreover, the direct interactions between two IR8a ATDs have been identified by protein fragment complementation assays (Abuin et al., 2019), suggesting that ATDs in coreceptors are involved in the assembly of heteromeric complexes. Tuning IRs and less broad coreceptor IRs, IR76b and IR93a, do not contain typical ATD domains (Figure 1) (Benton et al., 2009; Croset et al., 2010). Instead, they have a relatively short and highly divergent N-terminal region of about 200 amino acids before the LBD S1 domain (Croset et al., 2010; Abuin et al., 2011). Interestingly, deletion of the short amino acid N-terminal region in IR84a also abolishes the normal cilia targeting, suggesting that this fragment has a similar importance to the ATD domain in coreceptors in protein folding, IR complex assembly, or cilia targeting (Abuin et al., 2011). Moreover, the short amino acid N-terminal regions in two IR84a subunits do not interact directly (Abuin et al., 2019).

Both coreceptors and tuning receptors contain the ligand-binding domain (LBD) and transmembrane domain (TMD) (Figure 1) (Benton et al., 2009). The IR LBDs are highly divergent and, thus, the ligand-binding specificity of most or all IRs is likely to be both distinct from that of iGluRs and varied within the IR family (Benton et al., 2009; Croset et al., 2010; Abuin et al., 2011). For example, all iGluRs have an arginine (R) residue in S1 that binds the glutamate α-carboxyl group (C(=O)OH); only 19 IRs retain this residue. Moreover, a threonine (T) residue in the first half of S2 exists in all AMPA and kainate receptors and functions to contact the glutamate γ-carboxyl group. Only 9 IRs retain this residue. In the second half of S2, all iGluRs have an aspartate (D) or glutamate (E) that interacts with the glutamate α-amino group (NH_2_). Only 10 IRs retain this conserved residue. Importantly, only three IRs (IR8a, IR75a, and IR75c) retain all three conserved residues (R, T, and D/E). These conserved residues, at least in IR8a, are unlikely to recognize glutamate by the mutational analysis of IR8a (Mayer, 2006; Benton et al., 2009; Abuin et al., 2011).

The LBD domains are important for receptor targeting and chemical recognition. Mutation of the conserved R and D in IR8a reduces or abolishes the efficiency of targeting the cilia, suggesting a role for the IR8a LBD in receptor localization (Abuin et al., 2011). IR84a retains the R residue in the S1 domain. A point mutation of this residue does not affect the receptor targeting to cilia, but eliminates the ligand-evoked responses, indicating that the IR84a LBD is required for chemical recognition (Abuin et al., 2011). Interestingly, this R residue is conserved in acid-sensing IRs (IR31a, IR64a, IR75a, IR75b, IR75c, and IR84a) but divergent in amine-sensing IRs (IR41a, IR76a, and IR92a) (Box 1; Table 1), suggesting that this R residue might play a conserved role between iGluRs and acid-sensing IRs in conjugating the carboxyl group (C(=O)OH) of their ligands (Benton et al., 2009; Silbering et al., 2011). Studies on IR75a in different *Drosophila* species provide additional evidence about the function of the LBD domain in chemical recognition. Mutations of three amino acids (T289, Q536, and F538) in the IR75a LBD domain changes the responses to acetic acid, butyric acid, and 2-oxopentanoic acid (Box 2), suggesting one or more of these residues act as chemical-specificity determinants (Prieto-Godino et al., 2016).

Box 1 **Ester, alcohol, amine, and carboxylic acid.**

An **alcohol** carries at least one OH group bond to a saturated carbon atom.

An **amine** is a derivative of ammonia (NH_3_), wherein one or more hydrogen atoms have been replaced by a substituent.

A **carboxylic acid** contains a C(=O)OH group attached to an alkyl group. The amino acid and fatty acids are carboxylic acids.

An **ester** is a derivative of an acid in which at least one C(=O)OH group is replaced by a C(=O)OR group. R represents an alkyl group.

A **ketone** contains a C(=O) group bonded to two alkyl groups.

Box 2 **Chemical formulas mentioned in this review.**

Acetic acid: CH_3_C(=O)OH

Propionic acid: CH_3_CH_2_C(=O)OH

Butyric acid: CH_3_(CH_2_)_2_C(=O)OH

Hexanoic acid: CH_3_(CH_2_)_4_C(=O)OH

Octanoic acid: CH_3_(CH_2_)_6_C(=O)OH

Linoleic acid: CH_3_(CH_2_)_4_CH=CHCH_2_CH=CH(CH_2_)_7_C(=O)OH

2-Oxopentanoic acid: CH_3_(CH_2_)_2_C(=O)C(=O)OH

Lactic acid: CH_3_CH(OH)C(=O)OH

Tartaric acid: C(=O)OHCH(OH)CH(OH)C(=O)OH

Citric acid: C(=O)OHC(OH)(CH_2_C(=O)OH)_2_

Phenylacetic acid: C_6_H_5_CH_2_C(=O)OH

Methylamine: CH_3_NH_2_

Dimethylamine: CH_3_NHCH_3_

Trimethylamine: (CH_3_)_3_N

Ethylamine: CH_3_CH_2_NH_2_

Triethylamine: (CH_3_CH_2_)_3_N

Butylamine: CH_3_(CH_2_)_3_NH_2_

1,4-Diaminobutane: NH_2_(CH_2_)_4_NH_2_

Cadaverine: NH_2_(CH_2_)_5_NH_2_

Spermidine: NH_2_(CH_2_)_3_NH(CH_2_)_4_NH_2_

Pyridine: C_5_H_5_N

Pyrrolidine: (CH_2_)_4_NH

Phenethylamine: C_6_H_5_(CH_2_)_2_NH_2_

Phenylacetaldehyde: C_6_H_5_CH_2_C(=O)H

The coreceptor extra loop (CREL) is highly conserved in IR8a and IR25a across the insect and Protostomia, respectively. This loop is absent in tuning IRs or iGluRs (Figure 1). CRELs locate near the beginning of the S2 domain and possess several conserved characteristics, including the presence of predicted short alpha-helical and beta-sheet regions and a single consensus N-glycosylation target motif (NXS/T) (Abuin et al., 2019). Deletion of CREL or mutation of the N-glycosylation site in CREL affects the cilia trafficking of the IR complex, suggesting a role of CREL in receptor targeting (Abuin et al., 2019).

The ion channel pore is the most conserved region between IRs and iGluRs (Benton et al., 2009; Croset et al., 2010). To confirm that IRs act as ion channels, multiple point mutations in the putative ion channel pore have been generated. In the pore region of GluA2, glutamine is required to control calcium permeability (Hume et al., 1991). In the same position, IR84a retains glutamine (Q401). The IR84a mutant receptors that have a point mutation of this glutamine do not affect the conductance of monovalent cations but lack calcium permeability (Abuin et al., 2011; Ng et al., 2019). Moreover, IR8a contains a proline in the pore region. A point mutation of this proline decreases the ligand-evoked currents, affects the conductance of monovalent cations, and abolishes the calcium-dependent conductance (Abuin et al., 2011). These data together support the notion that IRs function as ion channels and suggest that the pore regions of both coreceptors and tuning receptors are required to mediate the ionic pathway.

TM3 is also involved in controlling the ion conductance of iGluRs (Sobolevsky et al., 2009). In the absence of ligands, iGluRs are closed. A spontaneous A288T mutation in TM3 disrupts the closed conformation, resulting in a constitutive sodium conductance (Zuo et al., 1997). When expressing in HEK293T cells, IR76b behaves as a sodium leaky channel and is in a constitutively open state. This constitutive current can be attenuated by a T to A point mutation in the corresponding position of IR76b (Zhang et al., 2013). This point mutation also blocks the *in vivo* low salt responses (Zhang et al., 2013).

While deletion of the CTD (Figure 1) of IR84a does not affect localization or function, deletion of the CTD of IR8a strongly reduces cilia-targeting efficiency (Abuin et al., 2011), suggesting a role of the coreceptor CTD domains in receptor localization.

Interestingly, when IR8a is the coreceptor in an IR complex, the complex often contains a single tuning receptor and, thus, two IR8a subunits and a couple of the same tuning receptors are in the complex (Abuin et al., 2011, 2019). However, IR25a often complexes with IR76b to mediate chemical sensation and IR93a to mediate temperature and humidity sensation (Table 1). Studies about the structure of IR complexes that include IR25a, IR76b, and IR93a have not been reported.

## Chemosensation

Animals mainly depend on their smell and taste to assess the quality and nutritional value of food. While olfaction plays a key role for insects to identify food, gustation is crucial for the decision of whether to feed. Moreover, both olfaction and gustation are important for courtship behavior (Squire et al., 2003; Montell, 2009; Carey and Carlson, 2011).

### Olfaction

**Figure 2:**
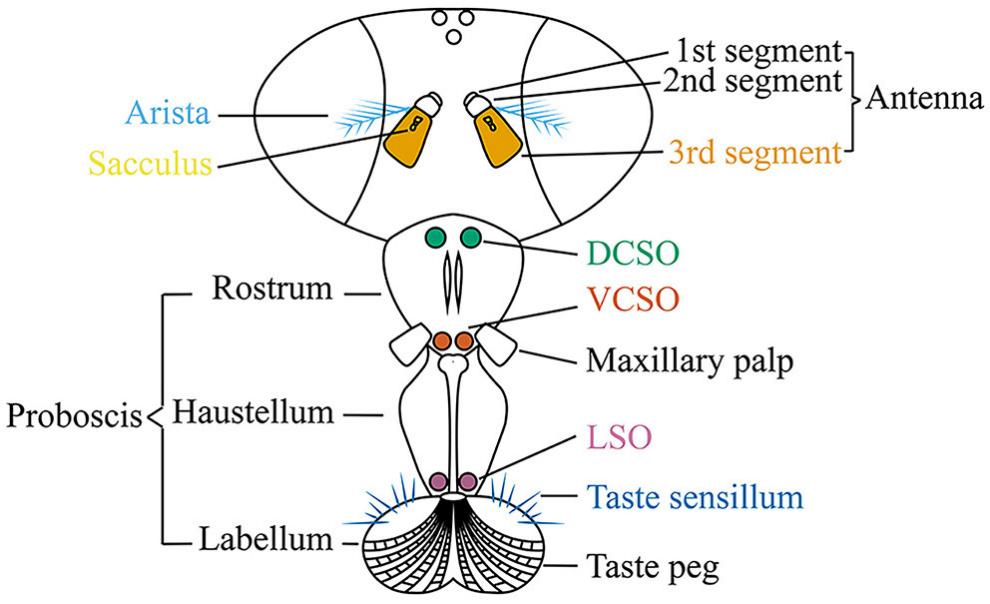
The adult fly head to show the olfactory organs (the third antennal segment and maxillary palp), gustatory organs [the labellar taste sensillum, labellar taste peg, labial sense organ (LSO), ventral cibarial sense organ (VCSO), and dorsal cibarial sense organ (DCSO)], thermosensory organ (the arista), and hygrosensory organ (the sacculus).

Olfactory sensory organs in adult flies are housed in the third antennal segment and maxillary palp (Figure 2). Each olfactory sensory neuron (OSN) is located in a sensillum and expresses a specific type or very small combination of receptors. All OSNs that express the same receptor converge their axons to a single pair of glomeruli in the antennal lobe (Vosshall and Stocker, 2007). Upon additional local processing at the level of the antennal lobe, the odor information is sent via projection neurons to two main higher brain centers, the mushroom body and the lateral horn (Masse et al., 2009).

The third antennal segment bears three classes of olfactory sensilla: basiconic, trichoid, and coeloconic sensilla. Each sensillum contains the dendrites of up to three OSNs with different odorant response profiles. Three classes of olfactory molecular receptors have been identified: the odorant receptors (ORs), ionotropic receptors (IRs), and a few gustatory receptors (GRs) (Vosshall et al., 2000; Jones et al., 2007; Benton et al., 2009). OSNs innervating basiconic and trichoid sensilla generally express ORs and respond to many esters, ketones, and alcohols; while, except for OR35a-expressing neurons, OSNs housed in coeloconic sensilla express IRs and respond to many amines and carboxylic acids (Box 1) (de Bruyne et al., 2001; Hallem et al., 2004; Yao et al., 2005; van der Goes van Naters and Carlson, 2007; Vosshall and Stocker, 2007; Benton et al., 2009; Silbering et al., 2011). Coeloconic sensilla can be classified into four clusters (ac1, ac2, ac3, and ac4) by their expression of stereotyped combinations of IR genes (Benton et al., 2009). IRs are also expressed in the sacculus, a three-chambered pit under the antennal surface, and in the arista, a feather-like structure that extended from the third segment of the antenna (Foelix et al., 1989; Shanbhag et al., 1995) (Figure 2). Although OR- and IR-expressing OSNs project to complementary sets of glomeruli in the antennal lobe, the axon terminals of the projection neurons postsynaptic to IR or OR OSNs are interdigitated within the mushroom body and the lateral horn (Silbering et al., 2011).

Three IR coreceptors, IR8a, IR25a, and IR76b, function in olfactory systems. IR8a is broadly expressed in the main portion of the third antennal segment and the third chamber of the sacculus (Supplementary Table 1) (Benton et al., 2009; Abuin et al., 2011; Ai et al., 2013; Menuz et al., 2014). IR8a is not detected elsewhere in the adult head, body, appendages, or larval stages (Benton et al., 2009; Sanchez-Alcaniz et al., 2018). IR8a is a coreceptor, generally forming heterotetrameric complexes with tuning IR receptors to detect carboxylic acids (Box 1) (Silbering et al., 2011). IR8a in mosquitoes is also necessary for sensing acidic volatiles, such as lactic acid, and human odor (Box 2) (Raji et al., 2019).

IR25a is the IR gene that is most similar to the ancestral IR and evolved from a bilaterian non-NMDA receptor gene (Croset et al., 2010). It is another coreceptor that is broadly expressed in the main portion of the third antennal segment (Benton et al., 2009; Abuin et al., 2011; Chen et al., 2015). Unlike IR8a, IR25a is expressed in the first and second saccular chambers and arista (Benton et al., 2009; Abuin et al., 2011; Chen et al., 2015; Steck et al., 2018; Budelli et al., 2019). IR25a is also found in the second antennal segment (Chen et al., 2015), proboscis (Chen and Amrein, 2017; Chen and Dahanukar, 2017; Lee et al., 2018; Sanchez-Alcaniz et al., 2018; Steck et al., 2018), legs (Chen et al., 2015; Chen and Amrein, 2017; Lee et al., 2018; Sanchez-Alcaniz et al., 2018; Steck et al., 2018), wings (Sanchez-Alcaniz et al., 2018), and abdomen (Sanchez-Alcaniz et al., 2018) in adults (Supplementary Table 1). In larvae, IR25a is broadly expressed (Supplementary Table 2) (Stewart et al., 2015; Croset et al., 2016; van Giesen et al., 2016; Rist and Thum, 2017; Sanchez-Alcaniz et al., 2018). As an olfactory receptor, IR25a detects amines when forming heterotetrameric complexes with tuning IR receptors (Box 1) (Silbering et al., 2011). Generally, IR25a and IR8a have selective functional properties and cannot substitute each other (Abuin et al., 2011).

IR76b is the third identified coreceptor in the olfactory system that is expressed in the main portion of the third antennal segment, but not the sacculus or arista (Benton et al., 2009; Menuz et al., 2014; Hussain et al., 2016; Steck et al., 2018). IR76b is also broadly expressed outside of the olfactory system, including the proboscis (Zhang et al., 2013; Hussain et al., 2016; Chen and Amrein, 2017; Chen and Dahanukar, 2017; Ganguly et al., 2017; Jaeger et al., 2018; Lee et al., 2018; Sanchez-Alcaniz et al., 2018; Steck et al., 2018), legs (Zhang et al., 2013; Hussain et al., 2016; Chen and Amrein, 2017; Ganguly et al., 2017; Lee et al., 2018; Sanchez-Alcaniz et al., 2018; Steck et al., 2018), wings (Zhang et al., 2013; Hussain et al., 2016; Sanchez-Alcaniz et al., 2018; Yanagawa et al., 2019), and abdomen (Supplementary Table 1) (Sanchez-Alcaniz et al., 2018). Moreover, IR76b is detected in larvae (Supplementary Table 2) (Stewart et al., 2015; Croset et al., 2016; Rist and Thum, 2017; Sanchez-Alcaniz et al., 2018). In olfactory systems, IR76b often complexes with IR25a to detect variant odorants. Unlike IR8a or IR25a, IR76b does not contain the ATD or CREL domain.

IR31a is expressed in the OSNs that project to antennal ac1 coeloconic sensilla (Benton et al., 2009). Their axons terminate at VL2p glomeruli in the antennal lobe (Silbering et al., 2011). The coreceptor, IR8a, is necessary for the function of IR31a neurons to respond to 2-oxopentanoic acid (Box 2; Table 1) (Silbering et al., 2011).

IR41a is expressed in the OSNs whose dendrites are housed in antennal ac2 coeloconic sensilla (Silbering et al., 2011). Their axons are projected to VC5 glomeruli (Silbering et al., 2011; Hussain et al., 2016). The IR41a positive OSNs respond to amine ligands, including pyridine, pyrrolidine, 1,4-diaminobutane (putrescine), cadaverine, and spermidine (Box 2) (Silbering et al., 2011; Hussain et al., 2016). IR25a and IR76b are coexpressed with IR41a and necessary for the amine responses (Table 1) (Abuin et al., 2011; Silbering et al., 2011; Hussain et al., 2016).

IR64a is expressed in the OSNs surrounding the third saccular chamber (Benton et al., 2009; Ai et al., 2010). These OSNs send their axons to two pairs of glomeruli, DC4 and DP1m (Ai et al., 2010, 2013; Silbering et al., 2011; Gao et al., 2015). The dendrites of DC4- and DP1m-targeting OSNs innervate ventral and dorsal compartments of the third saccular chamber, respectively (Ai et al., 2013). IR8a is expressed in both DC4- and DP1m-targeting IR64a positive neurons (Ai et al., 2013). DC4-targeting OSNs detect hydrochloric acid (HCl) and acetic acid (Box 2). IR8a and IR64a form an acid receptor that is necessary and sufficient for acid responses (Table 1) (Ai et al., 2010, 2013; Silbering et al., 2011; Gao et al., 2015).

IR75a is expressed in the ac2/3 coeloconic OSNs. The axons of ac2 OSNs target DP1l glomeruli, while ac3 axons target DL2 (Benton et al., 2009; Silbering et al., 2011; Prieto-Godino et al., 2016, 2017). IR75a forms an acidic receptor with IR8a and is necessary and sufficient to detect acetic acid and propionic acid (Box 2) (Abuin et al., 2011; Silbering et al., 2011; Gorter et al., 2016; Prieto-Godino et al., 2016). IR75b and IR75c are expressed in the ac3I and ac3II coeloconic OSNs, respectively (Prieto-Godino et al., 2017). Both IR75b and IR75c are sufficient to respond to propionic acid, butyric acid, and 2-oxyopentanoic acid (Box 2) with different sensitivities. These responses also depend on IR8a (Table 1) (Benton et al., 2009; Abuin et al., 2011; Silbering et al., 2011; Prieto-Godino et al., 2017).

IR75d is expressed in the ac1/2/4 coeloconic OSNs that send their axons to VL1 glomeruli (Benton et al., 2009; Silbering et al., 2011). IR75d-expressing OSNs respond to pyrrolidine and these responses are IR25a dependent (Box 2; Table 1) (Silbering et al., 2011).

IR76a is expressed in the ac4 coeloconic OSNs that project to VM4 (Benton et al., 2009; Silbering et al., 2011). IR76a and two coreceptors, IR25a and IR76b, are sufficient to detect pyrrolidine and phenylethylamine (Box 2; Table 1) (Abuin et al., 2011; Silbering et al., 2011).

IR84a is expressed in the ac4 coeloconic OSNs that send their axons to VL2a (Benton et al., 2009; Grosjean et al., 2011; Silbering et al., 2011; Hueston et al., 2016). Two IR8a subunits and two IR84a subunits form a heterotetramer that is necessary and sufficient to detect phenylacetic acid and phenylacetaldehyde (Box 2; Table 1) (Benton et al., 2009; Abuin et al., 2011; Grosjean et al., 2011; Silbering et al., 2011). VL2a is one of the only three glomeruli that are larger in males and whose inputs and outputs express male-specific isoforms of the behavioral sex determination gene fruitless (Manoli et al., 2005; Stockinger et al., 2005). Although IR84a does not show sexually dimorphic expression and is not tuned to fly-derived pheromones, it increases male courtship in the presence of IR84a ligands, such as phenylacetic acid (Grosjean et al., 2011).

IR92a is expressed in the ac1 coeloconic OSNs that projects to VM1 (Benton et al., 2009; Silbering et al., 2011). IR92a is necessary to detect ammonia, methylamine, dimethylamine, trimethylamine, ethylamine, triethylamine, butylamine, and pyrrolidine (Box 2; Table 1) (Silbering et al., 2011; Min et al., 2013). Although IR92a is found to be coexpressed with IR8a, IR25a, and IR76b, none of these coreceptors are necessary for the responses of ammonia or other amines (Abuin et al., 2011; Min et al., 2013).

IR25a and IR76b have been identified in the larval olfactory system, the dorsal organ ganglion (DOG) (Supplementary Table 2) (Stewart et al., 2015; van Giesen et al., 2016; Sanchez-Alcaniz et al., 2018). However, their functions in larval olfaction have not been demonstrated.

### Gustation

**Figure 3:**
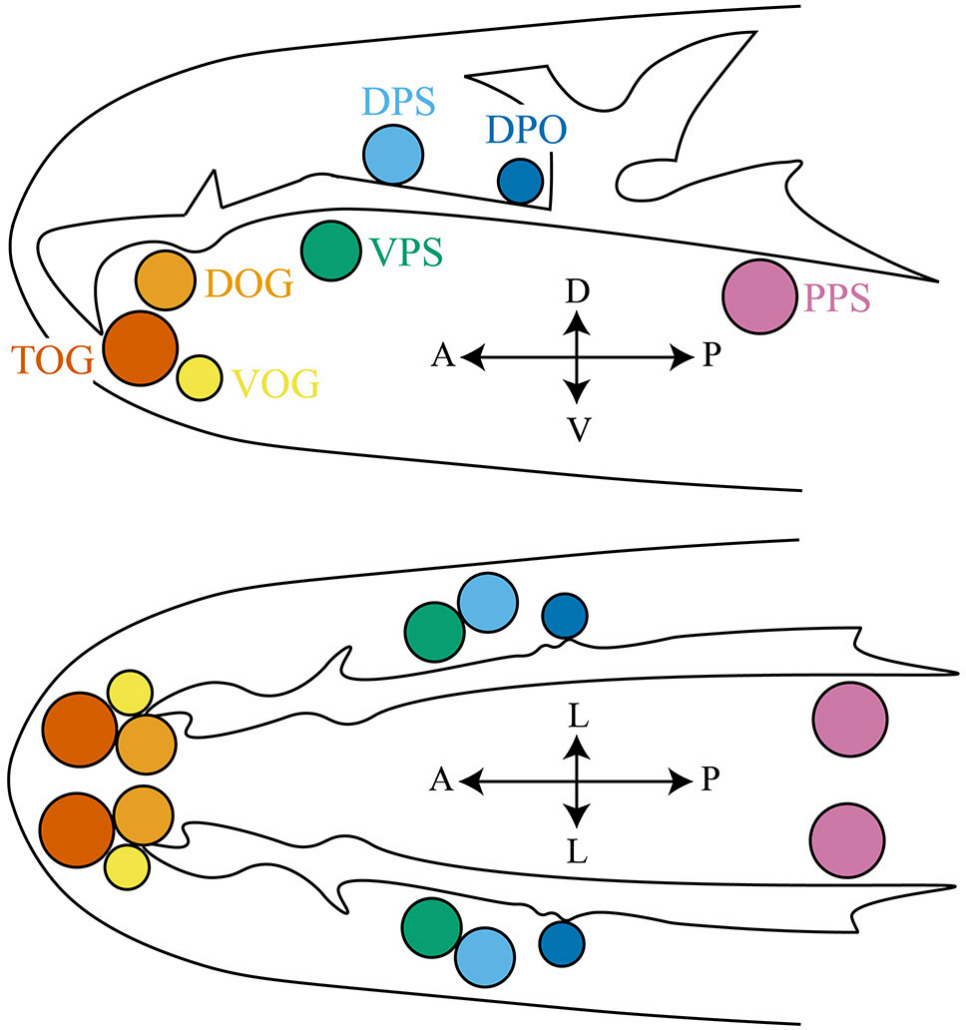
The larval anterior part to show the chemosensory organs, including the terminal organ ganglion (TOG), dorsal organ ganglion (DOG), ventral organ ganglion (VOG), dorsal pharyngeal sensilla (DPS), ventral pharyngeal sensilla (VPS), posterior pharyngeal sensilla (PPS), and dorsal pharyngeal organ (DPO). Larval thermosensitive cells are housed in the DOG. Upper panel: top view; lower panel: side view. The orientation is shown by double headed arrows. A, anterior; P, posterior; V, ventral; D, dorsal; L, lateral.

Gustatory receptor neurons (GRNs) in adult flies are found in many peripheral organs, including the labellum, pharynx, legs, wing margins, and abdomen (Figure 2). The labellum contains taste sensilla and taste pegs. The pharynx contains three organs: the labial sense organ (LSO), ventral cibarial sense organ (VCSO), and dorsal cibarial sense organ (DCSO). These pharyngeal taste organs are critical to monitor food quality. The head of the *Drosophila* larva contains three external chemosensory organs: the dorsal organ ganglion (DOG), terminal organ ganglion (TOG), and ventral organ ganglion (VOG) (Figure 3). In the pharynx, there are four internal chemosensory organs: the dorsal, ventral, and posterior pharyngeal sensilla (DPS, VPS, and PPS), and dorsal pharyngeal organ (DPO). Moreover, the posterior tip of the larva also contains chemosensory neurons. GRNs are housed in sensilla that have a single pore for tastants to enter (Montell, 2009). IRs have been found in all identified taste organs (Supplementary Tables 1, 2). Unlike IRs and ORs which are expressed in complementary OSNs, IRs and GRs are coexpressed in GRNs (Koh et al., 2014).

IR25a and IR76b, not IR8a, function as gustatory coreceptors to detect different tastants. They are broadly expressed in all taste organs in both larval and adult flies, with the exception that IR76b is not detected in the posterior tip of the larva (Supplementary Tables 1, 2) (Zhang et al., 2013; Chen et al., 2015; Stewart et al., 2015; Croset et al., 2016; Hussain et al., 2016; van Giesen et al., 2016; Chen and Amrein, 2017; Chen and Dahanukar, 2017; Ganguly et al., 2017; Rist and Thum, 2017; Jaeger et al., 2018; Lee et al., 2018; Sanchez-Alcaniz et al., 2018; Steck et al., 2018; Yanagawa et al., 2019). As in olfactory systems, IR25a and IR76b often collaboratively mediate responses to tastants. For example, both IR25a and IR76b are necessary to detect acids in tarsal GRNs, including acetic acid, citric acid, tartaric acid, hexanoic acid, octanoic acid, linoleic acid, and hydrochloric acid (Box 2) (Ahn et al., 2017; Chen and Amrein, 2017). In labellar taste bristles, IR25a and IR76b are necessary for responses to salt (high and low), potassium chloride, and calcium (Jaeger et al., 2018; Lee et al., 2018). They can also function independently in mediating responses to other tastants. For example, IR25a, not IR76b, is necessary to detect denatonium, a bitter tastant, in the C7 GRN in the TO (van Giesen et al., 2016). On the other hand, IR76b but not IR25a is necessary to detect the yeast/amino acid mixture in the tarsal bristles and larval TO (Croset et al., 2016; Ganguly et al., 2017). Labellar IR76b is necessary to detect polyamines, including 1,4-diaminobutane and cadaverine (Box 2), and to mediate avoidance of polyamines (Hussain et al., 2016). Moreover, IR76b alone is sufficient to form the low salt receptor that does not require additional IRs (Zhang et al., 2013). IR76b in the labellum has also been proposed as a sensitivity modulator to regulate the preference for acetic acid, citric acid, and sucrose (Box 2) (Chen H. L. et al., 2019).

IR7a is expressed in the bitter GRNs in the labellum and is required for the avoidance of acetic acid (Box 2) (Rimal et al., 2019). Although IR25a is coexpressed with IR7a, it is not necessary for the function of IR7a in detecting acetic acid (Table 1) (Croset et al., 2010; Rimal et al., 2019). IR7a and IR7f are expressed in the blood-sensitive GRNs on the stylet tip of female mosquitoes (Jové et al., 2020).

IR20a in tarsal GRNs is coexpressed with IR76b and, at least partially, required for detecting amino acid mixture (Table 1) (Ganguly et al., 2017). Since the loss of *IR20a* results in weaker defects in amino acid detection, IR20a may be functionally redundant with other IRs (Ganguly et al., 2017). Moreover, expression of IR20a is sufficient to confer sensitivities of amino acid mixture to labellar sensilla and to block the function of IR76b in detecting low salt (Zhang et al., 2013; Ganguly et al., 2017).

IR52a is expressed in the adult legs and wings (Koh et al., 2014; He et al., 2019). It shows sexual dimorphic expression and is necessary for sexual behavior in both male and female flies (Koh et al., 2014; He et al., 2019). IR52c and IR52d are expressed in sexually dimorphic taste neurons in forelegs. These neurons are activated by contacting females during courtship (Koh et al., 2014). The function of IR52a, IR52c, and IR52d may depend on IR25a and IR76b, since IR25a and IR76b are coexpressed with IR52a, IR52c, and IR52d in forelegs (Koh et al., 2014). However, the chemical cues that activate IR52a, IR52c, and IR52d have not been found.

IR56d is found in labellar taste sensilla and pegs, as well as tarsal bristles (Ahn et al., 2017; Tauber et al., 2017; Sanchez-Alcaniz et al., 2018). Together with IR25a and IR76b, IR56d is necessary for the responses to multiple fatty acids, including hexanoic acid, octanoic acid, and linoleic acid (Box 2; Table 1) (Ahn et al., 2017; Tauber et al., 2017; Sanchez-Alcaniz et al., 2018). Interestingly, the *norpA* gene, which encodes a phospholipase C (PLC) that acts as a downstream effector of G-protein coupled receptors in the fly's visual system, is necessary for these responses (Ahn et al., 2017; Tauber et al., 2017). These three IRs are also necessary to detect carbonations in taste pegs (Table 1) (Sanchez-Alcaniz et al., 2018).

IR60b mediates sucrose responses in LSO in the adult pharynx and acts in limiting sucrose consumption (Table 1) (Koh et al., 2014; Joseph et al., 2017; Chen Y. D. et al., 2019).

IR62a forms a calcium-responsive receptor with IR25a and IR76b in the labellum and is necessary to mediate calcium avoidance (Table 1) (Lee et al., 2018).

## Thermosensation and Hygrosensation

Temperature and humidity influence the fitness and geographic distribution of all animals and are crucial for insects. Insects rely on the ambient temperatures to set their body temperatures and must avoid dehydration, a constant threat for insects due to their small bodies and large ratios of surface area to volume (Garrity et al., 2010; Chown et al., 2011).

IRs function in thermosensation and hygrosensation. In adult flies, IR-formed thermoreceptors have been identified in the arista and sacculus (Figure 2), although the molecular basis of the saccular thermoreceptors has not been resolved (Enjin et al., 2016; Frank et al., 2017; Budelli et al., 2019). The sacculus also houses hypersensitive neurons that depend on IRs to detect the humidity change (Enjin et al., 2016; Knecht et al., 2016, 2017; Frank et al., 2017). While the IR-formed humidity receptors have not been found in larvae, the IR-formed thermoreceptors have been identified in the larval dorsal organ ganglion (DOG) (Figure 3) (Knecht et al., 2016; Ni et al., 2016).

As discussed, IR25a is broadly expressed and functions as a coreceptor for olfaction and gustation to detect variant chemicals. In addition, IR25a is also expressed in thermoresponsive neurons in the larval DOG and adult arista to support thermosensation, as well as humidity sensitive neurons in the sacculus to support hygrosensation (Figures 2, 3; Supplementary Tables 1, 2) (Benton et al., 2009; Enjin et al., 2016; Knecht et al., 2016, 2017; Ni et al., 2016; Frank et al., 2017; Budelli et al., 2019). Moreover, IR25a in the femur chordotonal organ neurons is proposed to be a warm receptor to mediate circadian clock resetting by temperature, although the tuning receptors, if any, for this process are still unknown (Chen et al., 2015). In the brain, IR25a-dependent sensory neurons with different sensory modalities send their projections to different regions where the specific sensory information is processed. For example, the antennal IR25a-positive sensory neurons project to distinct glomeruli based on the specific tuning receptors. The IR41a- and IR76a-dependent OSNs send their axons to two different glomeruli, VC5 and VM4 (Benton et al., 2009; Silbering et al., 2011; Hussain et al., 2016). The aristal IR21a cool cells project to the VP3 glomerulus (Silbering et al., 2011; Marin et al., 2020). The VP4 and VP5 glomeruli receive information from IR40a dry cells and IR68a moist cells, respectively (Enjin et al., 2016; Knecht et al., 2016, 2017; Frank et al., 2017; Marin et al., 2020). In contrast, the IR25a-positive GRNs project to the sub-esophagael zone (SEZ), the primary taste center of the *Drosophila* central nervous system (Tauber et al., 2017; Sanchez-Alcaniz et al., 2018). Besides IR25a, IR93a also functions as a coreceptor specific for thermosensation and hygrosensation (Enjin et al., 2016; Knecht et al., 2016, 2017; Frank et al., 2017; Budelli et al., 2019).

The tuning receptor for cool detection is IR21a (Ni et al., 2016; Budelli et al., 2019). IR21a is expressed in the larval DOG and adult arista (Figures 2, 3). It forms a cool-active receptor with IR25a and IR93a and is necessary and sufficient to mediate the cool responsiveness (Table 1) (Ni et al., 2016; Budelli et al., 2019). These three IRs are also critical for the morphogenesis of the membrane-riched dendritic structure in cooling cells (Budelli et al., 2019). Although IR21a is expressed in the sacculus (Benton et al., 2009), the coreceptors and functional importance have not been identified. Moreover, IR21a in mosquitoes is necessary for host-seeking behavior (Greppi et al., 2020).

IR40a and IR68a are the tuning receptors for dry and moist detection, respectively (Enjin et al., 2016; Knecht et al., 2016, 2017; Frank et al., 2017). IR40a is expressed in the first and second saccular chambers and necessary for the dry activation of dry neurons (Figure 2; Table 1) (Benton et al., 2009; Enjin et al., 2016; Knecht et al., 2016). IR68a is mainly expressed in the second chamber of the sacculus and necessary for detecting moist air (Figure 2; Table 1) (Frank et al., 2017; Knecht et al., 2017). The function of IR40a and IR68a also depends on IR25a and IR93a (Table 1) (Enjin et al., 2016; Knecht et al., 2016, 2017; Frank et al., 2017).

## Discussion

In *Drosophila melanogaster*, 63 IRs have been identified and function in olfaction, gustation, thermosensation, and hygrosensation (Benton et al., 2009; Koh et al., 2014; van Giesen and Garrity, 2017). The expression of most IRs–except for IR48a, IR51a, IR54a, IR60a, and IR87a–has been shown by *in situ* hybridization, antibody staining, and/or driver lines (Supplementary Tables 1, 2). Besides four coreceptors (IR8a, IR25a, IR76b, and IR93a), the functions of only 19 tuning receptors have been identified (Table 1). IR52a, IR52c, and IR52d are expressed in taste neurons in wing margins and legs. They are required for courtship behavior (Koh et al., 2014; He et al., 2019). However, the ligands of these IRs are unknown and, thus, are not listed in Table 1. To comprehensively understand the IR family and peripheral sensory systems in *Drosophila melanogaster*, it is important to identify the function of each IR. Moreover, the IR family is a highly divergent subfamily of iGluRs (Benton et al., 2009). Although iGluRs have been well-studied, it is not fully understood how IR-formed receptors are gated by their chemical ligands. Most importantly, how do IR-formed receptors sense temperature and humidity change?

In addition, a tuning receptor usually binds one or two coreceptors to detect a specific environmental stimulus. However, although none of IR8a, IR25a, and IR76b is necessary, IR92a-mediated responses of ammonia can only be detected when IR92a is expressed in IR OSNs, but not OR OSNs, in the antenna (Abuin et al., 2011; Min et al., 2013), suggesting these three coreceptors might have redundant functions in supporting the IR92a-dependent responses of ammonia. Thus, it is worth testing whether a tuning IR can pair with different coreceptors to form a functional receptor and, if so, whether these receptors have the same function.

IRs are conserved across protostome species and bioinformatic analyses of *IR* genes have been performed in many species (Benton et al., 2009; Croset et al., 2010; Bengtsson et al., 2012; Glaser et al., 2013; Poivet et al., 2013; Rytz et al., 2013; Cao et al., 2014; Groh-Lunow et al., 2014; Liu et al., 2014, 2017, 2018; Missbach et al., 2014; Ahmed et al., 2016; Dippel et al., 2016; Macharia et al., 2016; van Schooten et al., 2016; Cicconardi et al., 2017; Latorre-Estivalis et al., 2017; Yang et al., 2017, 2020; Zbinden et al., 2017; Harrison et al., 2018; Kozma et al., 2018; Matthews et al., 2018; Robertson et al., 2018, 2019; Rojas et al., 2018; Andersson et al., 2019; Lei et al., 2019; Li et al., 2019; Ma et al., 2019; Xu et al., 2019; Balart-García et al., 2020; Sun et al., 2020; Wu et al., 2020; Yin et al., 2020). However, the functional studies of IRs in non-model organisms are largely lacking. This is mainly due to the limitation of genetic approaches in these organisms. To overcome this problem, two genetic approaches have been applied. First, *IR* genes from non-model organisms are cloned and expressed in *Drosophila melanogaster* to perform functional studies. For example, the functional specificities of *Drosophila sechellia* IR75a, IR75b, and IR75c are firstly obtained by expressing these genes in *Drosophila melanogaster* (Prieto-Godino et al., 2016, 2017). Moreover, expression of the mosquito IR76b is sufficient to rescue the defects in detecting amino acid mixture in *Ir76b* mutant flies (Ganguly et al., 2017). The IR25a and IR93a orthologues in honey bee parasitic mites, *Tropilaelaps mecedease*, are sufficient to rescue the temperature and humidity preference defects when expressing in *IR25a* and *IR93a* mutant flies, respectively (Lei et al., 2019). These data suggest conserved roles of IR76b, IR25a, and IR93a in sensory function, although the endogenous function is not evaluated. Second, with the advance of CRISPR techniques, genetic manipulation is achieved in many non-model organisms. For instance, the functional necessity of IR8a, IR75b, and IR64a in noni attraction is studied in *Drosophila sechellia* by generating the corresponding mutants using CRISPR techniques (Auer et al., 2020). In mosquitoes, the *IR21a* mutants have been generated by CRISPR techniques and its necessity for cool responses in thermosensory neurons in the antennal tip and heat-seeking behavior has been identified (Greppi et al., 2020). The *IR8a* mutant mosquitoes are also generated using CRISPR techniques. These mutant mosquitoes are fundamental to identify the necessity of IR8a in sensing human odor during blood feeding (Raji et al., 2019). The driver lines of *IR7a* and *IR7f* are expressed in the blood-sensitive GRNs on the stylet tip on female mosquitoes, although their contribution to blood ligand detection is unknown (Jové et al., 2020). Functional studies of IRs in different animals will play a key role in understanding molecular mechanisms of multiple sensory modalities in various disease vectors and pests and, thus, help to develop tools to control them.

## Author Contributions

LN drafted the initial manuscript and approved the final version of the manuscript.

## Conflict of Interest

The author declares that the research was conducted in the absence of any commercial or financial relationships that could be constructed as a potential conflict of interest.
